# Postnatal Depression Risk Factors: An Overview of Reviews to Inform COVID-19 Research, Clinical, and Policy Priorities

**DOI:** 10.3389/fgwh.2020.577273

**Published:** 2020-10-22

**Authors:** Frances Lee Doyle, Louis Klein

**Affiliations:** ^1^School of Psychology, Faculty of Science, The University of Sydney, Sydney, NSW, Australia; ^2^School of Psychiatry, University of New South Wales, Sydney, NSW, Australia; ^3^Mental Health Academic Unit, Liverpool Hospital, Sydney, NSW, Australia

**Keywords:** review, COVID-19, postpartum, postnatal, perinatal, depression, systematic, pandemic

## Abstract

The disruption of normal life due to the COVID-19 pandemic is expected to exacerbate extant risk factors for mental health problems. This may be particularly true for women who give birth during the crisis, especially those at risk for postnatal depression. Maternal postnatal depression has been identified as a public health issue with profound impacts on maternal and child well-being. Evidence from previous crises (e.g., earthquakes, terrorist attacks) has shown that crises significantly impact maternal mental health and some perinatal health outcomes. The aims of this paper were therefore to conduct a review to identify the established risk factors for maternal postnatal depression, and generate evidence-based hypotheses about whether the COVID-19 crisis would likely increase or decrease postnatal depression rates based on the identified risk factors. Several databases were searched during May-June 2020 for review papers (i.e., systematic reviews, meta-analyses, qualitative syntheses) using the following keywords: Depression, perinatal, postnatal, postpartum, systematic, review, predictors. Risk factors were extracted in conjunction with indicators for their strength of evidence (i.e., effect sizes, qualitative coding). Risk factors were critically evaluated in relation to their susceptibility to the impacts of the COVID-19 crisis. It was hypothesized that several health policies that were necessary to reduce the spread of COVID-19 (e.g., required restrictions) may be simultaneously impacting a range of these known risk factors and placing a larger number of women at heightened risk for postnatal depression. For instance, factors at a strong risk of being exacerbated include: Perceived low social support; exposure to traumatic events during or prior to pregnancy; significant life events occurring during pregnancy; and high stress associated with care of children. Future research and policy implications are discussed, including how policy makers could attempt to ameliorate the identified risk factors for postnatal depression following the current COVID-19 pandemic.

The novel coronavirus (COVID-19) pandemic is an unprecedented global event that has a range of health, economic, and socio-emotional implications. Understandably, governments are first and foremost addressing the physical health crisis, before responding to the economic implications. It is becoming apparent, however, that there is also a need to consider the mental health of vulnerable populations during and following the COVID-19 pandemic (1). Although postnatal depression has been identified as a major public health problem (2) with prevalence rates ranging from 10 to 40% globally (3), the mental health needs of women in the perinatal period have not yet been adequately considered in the context of the COVID-19 crisis. Evidence from previous crises highlights that perinatal health and maternal mental health tends to be negatively impacted in the wake of these crises (4, 5). It is therefore increasingly apparent that health policies necessary to reduce the spread of COVID-19 (e.g., required restrictions; changes to hospital policies; physical distancing; sheltering in-place; restricted travel) may also be simultaneously impacting a range of known risk factors for postnatal depression and thereby placing a larger number of women at heightened risk for postnatal depression.

## The COVID-19 Context as it Relates to Women in the Perinatal Period

Early research into the COVID-19 pandemic is beginning to reflect that the socio-emotional impacts are not universal (6). In this next section, an outline of how the COVID-19 crisis may relate to women during the perinatal period is provided to demonstrate impacts on this unique population. It is possible that home isolation and physical distancing may be associated with feelings of loss and loneliness as mothers' social supports are different from what they may have expected. For instance, home isolation and physical distancing measures may mean that new mothers are unable to have family members and friends support them following the birth of their child. This may include those who can no longer travel from overseas, those living across the country, as well as supports who may live physically closer but who are unable to visit due to physical distancing precautions (e.g., older adults). Loneliness may be also experienced, as physical distancing measures have required many postnatal supports to cease operating (e.g., postnatal mothers' groups; libraries; cafes; “mom's and bubs” gym classes); reducing the options for mothers to connect for social and practical support. In addition, some mother-infant dyads may not be able to establish, and maintain breastfeeding due to measures of isolation or separation guidelines. Breastfeeding duration has been associated with less postnatal depression and/or the amelioration of depression symptoms (7), thus some of these women may be at greater risk for postnatal depression.

Uncertainty around health risks (e.g., impact of COVID-19 on pregnancy outcomes) and changes to healthcare systems may also impact well-being during the perinatal period. Common experiences may include: Reduced numbers of support birth partners or no birth partners allowed into the birthing suite (8); reduced stay in hospitals after birth; concerns about management of COVID-19 patients within the same hospital facility; reduced or delayed help-seeking throughout pregnancy due to concerns about contracting COVID-19 when attending appointments; fewer in-person antenatal appointments reducing the frequency of checking mother and infant vital signs; and/or, separations of newborns from COVID-19 positive mothers for 14 days (9). Moreover, the availability of informational affordances for pregnant women and new mothers may also have been negatively affected, for example by canceling face-to-face antenatal/postnatal education classes. Reduced frequency of antenatal medical and sonogram appointments may further reduce opportunities for women to access timely information directly from healthcare professionals to reduce uncertainty and fears about health risks. Fewer antenatal appointments may also reduce opportunities for healthcare professionals to promote knowledge about a range of positive health behaviors for mothers and infants (e.g., positive benefits of breastfeeding, importance of mothers noticing any reduction in movements in the third trimester).

Even while the long-term economic impacts of COVID-19 are not yet known, short-term impacts (e.g., reduced wages; being laid-off; fewer rostered hours; lack of security associated with rostered work) are likely to increase general stress levels. Further, it is currently unclear how the emotional experience of financial hardship may be addressed or ameliorated with the intervention of government support packages. It is clear, however, that the impacts of financial hardship and the associated stress will likely be greatest for women without a spousal partner (10), recent (economic) migrants, asylum seekers undergoing resettlement processing, and refugees (11–13), and, women from traditional and/or conflict-affected backgrounds (14); the majority of whom do not have surplus financial reserves to draw upon during the crisis.

Stress from additional domestic caring duties during the COVID-19 crisis may also impact mothers. Research has shown that a greater number of hours of caring duties typically fall to women (15), and women may be juggling managing older children's educational and emotional needs alongside caring duties for older adults who are self-isolating. With families contained to their homes for extended periods, relationships may also experience strain. The perinatal period has been shown to have a high domestic and family violence risk (16), it is therefore possible that with additional strain comes additional violence risk for some women; particularly those who a history of victimization by their partners or who experience (or have partners that experience) substance-use disorders (17). Less contact with those from outside of the household (including medical and sonographer appointments) means it is possible that those women who experience acute stress from increased threat from, and experience of, domestic and family violence may have difficulty in attaining support (17).

### Evidence From Previous Crises

A number of systematic reviews have been conducted examining the effects of previous crises on perinatal health and mental health (4, 5, 18). After reviewing studies examining terrorist attacks (e.g., September 11), environmental and chemical disasters (e.g., nuclear reactor accidents at Chernobyl), and natural disasters (e.g., hurricanes, earthquakes, floods), Harville, Xiong (4) found that severity of exposure to the crisis/disaster was a major risk factor for poor mental health outcomes among pregnant and postpartum women. Further, Harville, Xiong (4) concluded that following crises/disasters, mothers' mental health may more strongly influence child development than any direct effects of crisis/disaster-related prenatal stress. Ren, Chiang (5) examined the mental health of pregnant women following earthquakes and, while they could not determine whether postnatal depression rates were increased, they found that antenatal depression rates were more prevalent in women who had experienced an earthquake during pregnancy than those who had not. Finally, Saulnier and Brolin (18) concluded that maternal stress was a common underlying determinant of children's long-term health when the child was exposed to crises during pregnancy. These reviews on previous crises thus highlight the importance of considering women's mental health in the postnatal period following the COVID-19 pandemic.

Moreover, it is evident that there may be interplay between factors that are indirectly influenced by crises and women's mental health (17). For instance, research has shown that domestic and family violence reports have peaked following previous crises (e.g., the eruption of Mount St. Helens in the U.S.A., 1982; Hurricane Katrina, 2005; “Black Saturday” bushfires in Australia, 2009; Haitian earthquake, 2010) and continued to occur at increased rates for at least a year following crises (17, 19–23). Therefore, it is possible that certain crises may inflate some indirect relationships more than others.

### Emerging Evidence From the COVID-19 Pandemic

Early evidence has shown pregnant women are not at a greater risk of catching COVID-19 than the general population (24, 25). However, evidence from other respiratory infections shows that pregnant women may be at risk of greater harm if they get a respiratory infection (24, 26, 27). Particularly during the COVID-19 crisis, it is probable that not all women in the antenatal and postnatal periods will have equal access to this information. The importance of access to official healthcare information has been reiterated by research conducted in China in the early stages of the COVID-19 pandemic (28). Findings indicated that pregnant women who had not accessed antenatal health information from hospitals' official social media accounts self-reported significantly higher stress, anxiety, and depressive symptoms than those who had (28). This study also uncovered that pregnant women in China during the early months of the pandemic were reporting higher rates of general symptoms of psychopathology than earlier cohorts (28). A finding that has been echoed by Davenport, Meyer (29) who conducted a rapid response survey in April-May 2020 capturing data from 900 predominantly North American women in the antenatal and postnatal periods where high levels of self-reported depression and anxiety symptoms were found (29). Thus, early evidence is indicating that higher rates of mothers' mental health symptoms are emerging and are not country-specific.

### The Current Study

There are a range of key risk factors that need to be considered when planning how to support and provide interventions to ameliorate the socio-emotional impacts on women and their children during, and following, the COVID-19 global pandemic crisis. Evidence from crises suggests that some risk factors will be more negatively affected than others; thus, we are hypothesizing that there will be an increase in the population prevalence of postnatal depression following the COVID-19 crisis period. In order to identify the range of risk factors most vulnerable to impacts of the COVID-19 crisis, the primary aim of this paper was to review the available summary evidence (i.e., systematic reviews, meta-analyses, qualitative syntheses) to determine a list of established risk factors for postnatal depression. Following this, the secondary aim of this paper was to provide hypotheses about whether the COVID-19 context would likely increase or decrease the identified risk factors for postnatal depression in women. In undertaking this analysis, we hope to equip mental health clinicians, researchers, and relevant policy makers to more effectively address maternal and antenatal mental health concerns following the COVID-19 pandemic. Finally, we hope to contribute to the growing evidence-base for the trade-offs public health settings make with women's mental health in times of crisis.

## Method

### Search Strategy

Identification of articles for this review was guided by the principles as outlined in the PRISMA (Preferred Reporting Items for Systematic Review and Meta-Analyses) statement (30). Accordingly, the following electronic databases were searched: SCOPUS, PubMed, EMBASE, PsycINFO, and the Cochrane Library. Each database was searched from its start date through to June 01, 2020 using the following keywords and their conjugates: Depression, perinatal, postnatal, postpartum, review, systematic, predictors. For example, in SCOPUS the search strategy was implemented using the following query: TITLE-ABS-KEY((depressi^*^ AND (perinatal OR postnatal OR postpartum)) AND (systematic OR review) AND (predicto^*^)) AND (LIMIT-TO (LANGUAGE, “English”)). These search terms were developed using an iterative strategy to ensure a high degree sensitivity to target literature. Additional articles were identified by combing the reference lists of relevant articles that met inclusion criteria, in addition to search of gray literature using Google Scholar. Searches were conducted by LK in close consultation with FD; both of which have extensive experience performing literature reviews. Only published peer-reviewed articles available in English were considered for this review.

### Article Selection Process

Peer-reviewed publications were identified in the initial stage of the search process with 338 potentially relevant titles, abstracts, and keywords. Each candidate was then evaluated according to the following predetermined exclusionary criteria: (a) The article focussed on factors primarily associated with paternal rather than maternal depression; (b) the article did not use depression and/or depressive symptoms as an explicit variable in analyses; (c) the article did not claim to report on risk factors of maternal depression; (d) analyses used depression and/or depressive symptoms to predict an exogenous factor not of interest; (e) the article was not relevant to the query (i.e. immediate exacerbation linked to the COVID-19 crisis) including those articles reporting on genetic factors, biomarkers, and endocrine factors associated with maternal depression; (f) the article was not a review, meta-analysis, or qualitative synthesis and therefore did not aggregate, or otherwise pool, data from multiple studies. Following the application of these exclusionary criteria, 27 articles were selected for further evaluation. During this process, 13 articles were further excluded according to the following additional exclusionary criteria: (g) The article did not adequately report on their review methodology, in accordance with Downs and Black (31), or, in the case of narrative reviews where these elements may not have been reported (32, 33), that the narrative treatment of reference literature was insufficiently rigorous; (h) The article did not report upon individual risk factors identified for maternal depression in sufficient detail for the purposes of the current review; (i) The article did not evaluate the degree of risk associated with reported risk factors (i.e., effect sizes; clinical risk frameworks). Both FD and LK reviewed article exclusions according to these outlined criteria.

## Results

**Figure 1:**
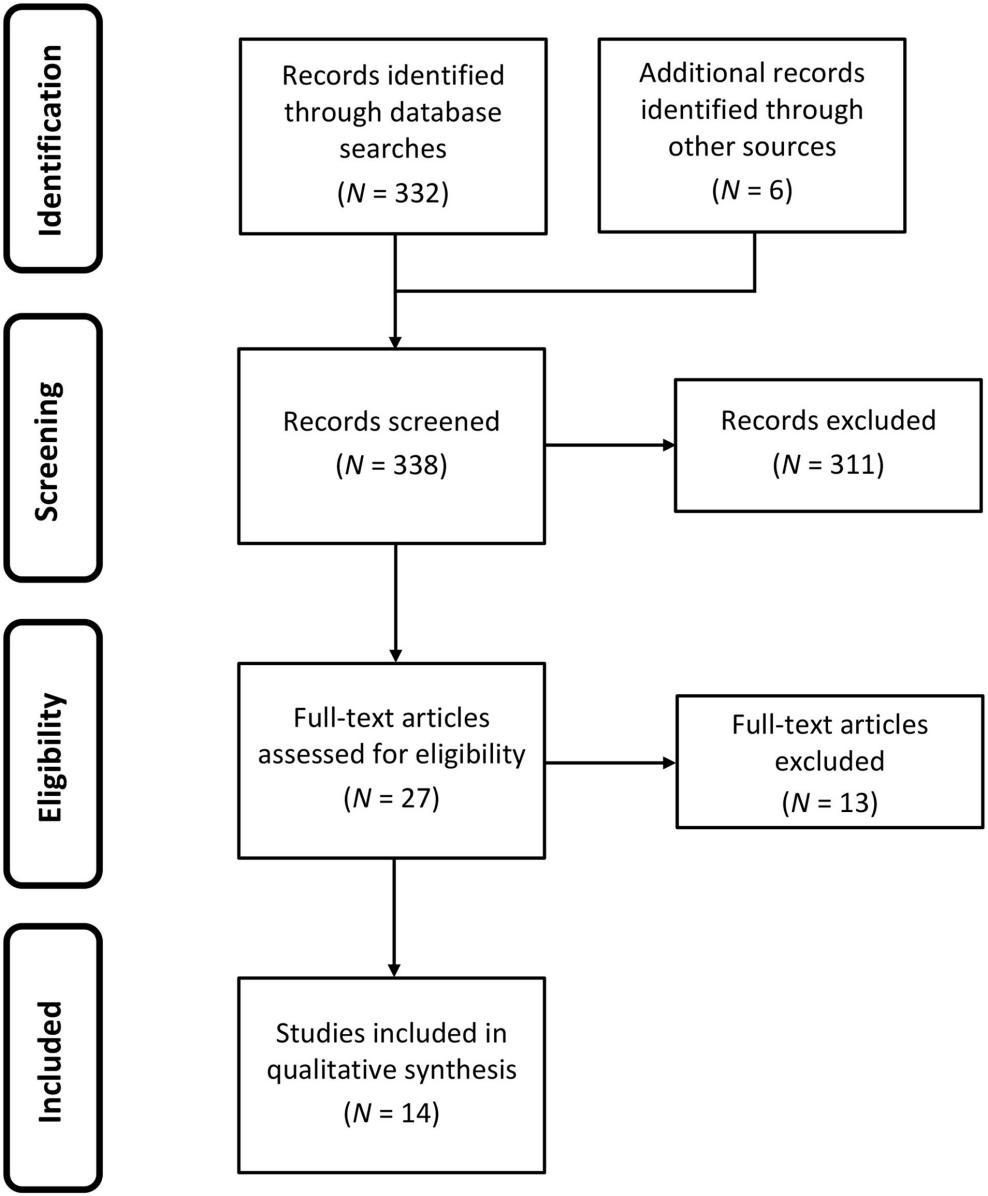
Flowchart reporting the identification and selection of studies for review and qualitative synthesis.

The article selection process yielded 14 articles included for review (for a flowchart of the article selection process see Figure 1 and for a summary of the included articles see Table 1): Eight articles were systematic reviews in which four of these eight explicitly conducted meta-analyses (35, 39–41); six articles used a narrative/synthetic approach to literature review. Nine of the 14 articles studied maternal depression in the general population, however five focussed upon features of maternal depression specific to the local demographic context (36, 40, 42, 43, 45). The most commonly searched databases within the articles, in order from highest frequency, were: PUBMED, Medline, PsycINFO, CINAHL; five articles did not report on the databases used to conduct their review or meta-analyses. The most common reason for the exclusion of articles was (f) accounting for ~36% of excluded articles; followed by (b) accounting for ~25%; and, (e) accounting for ~21%. Strength of evidence was determined by the following effect size thresholds (Cohen's *d*) (46): Strong overall evidence, *d* > 0.6; moderate overall evidence, 0.4 < *d* <0.6; weak overall evidence, *d* <0.4. In cases where effect sizes were unavailable, under-reported or not reported, an evaluation was made regarding the strength and diversity of domain-specific literature adduced in support of reported risk factors. Data quality overall was moderate to weak, although this can be partially attributed to the wide search window leading to the inclusion of several studies that predate modern statistical reporting standards. Due to this and the diversity of review methods employed across included articles, planned quantitative analyses including meta-analysis and effect size analysis could not be performed. However, a qualitative approach to the abstraction of risk factors of postpartum depression was undertaken with 25 core risk factors being identified (see Table 2). As displayed in Table 3, each risk factor for postnatal depression was then evaluated for whether risks were likely to be increased or decreased in the COVID-19 context. These hypotheses were determined by the authors based on the literature reviewed on crises, the observations about the context-specific contextual factors for pregnant women during COVID-19, and clinical judgment. FD independently coded each risk factor, and LK subsequently reviewed ratings. Author agreement was established for categorisations for all risk factors.

**Table 1:** Details of the studies included for review.

**Article short name**	**Review type**	**Databases searched in each article**	***N***	**Keywords**	**Demographic focus**
Banti et al. (34)	Narrative/synthetic	PUBMED		Pregnancy, perinatal depression, risk factors, clinical presentation, drug treatment	General
Beck (35)	Meta-analysis	CINAHL, MEDLINE, PsycINFO, Eric, Popline, Social Work Abstract, Sociological Abstracts, Dissertation Abstracts, JREF	84	Postpartum depression, postnatal depression, puerperal depression, predictors, risk factors	General
Cutrona (32)	Narrative/synthetic	Nil reported			General
Guintivano et al. (33)	Narrative/synthetic	Nil reported			General
Gulamani et al. (36)	Narrative/synthetic	CINAHL, ScienceDirect, MD Consult		Infants, mother, PPD, postpartum blues, post-natal depression, mental health, postpartum, preterm delivery, preterm infant(s), mother-infant interaction, mother-infant dyad, mother infant bonding, parental stress, early parental stress, culture, ethnicity, society	Pakistan
Koirala and Chuemchit (37)	Systematic	PUBMED, SCOPUS, Web of Science, Google Scholar	38	postpartum, postnatal, depression, violence	General
Lee and Chung (38)	Narrative/synthetic	Nil reported			General
O'Hara and Swain (39)	Meta-analysis	Nil reported			General
Özcan, Boyacioglu (40)	Meta-analysis	PUBMED, Science Direct, MEDLINE, PsycINFO, Ovid, CINAHL, Cochrane	52	Postpartum, puerperal, postnatal, depression, Turkey	Turkey
Robertson et al. (41)	Meta-analysis	Nil reported			General
Schmied et al. (42)	Systematic	SCOPUS, MEDLINE, PsycINFO, Health Source	23	Longitudinal, women, women's health, pregnancy, psychosocial, mental health, physical, infant, perinatal, postnatal	Australia, New Zealand
Takegata et al. (43)	Systematic	CINAHL, MEDLINE, PUBMED, Ovid, SCOPUS, IndMED, ICHUSI	50	Antenatal depression, postpartum depression, India, Japan	India, Japan
Yim et al. (44)	Systematic	PUBMED, PsycINFO	214	Postpartum, postnatal, social, psychosocial, endocrine, partner, immune, inflammatory, cytokine, genetic, stress, demands, events, couple, relationship, partner, marital, marriage, close relationship, interpersonal, social, family, social network, social support, integration	General
Zahidie and Jamali (45)	Narrative/synthetic	PUBMED	12	Depression, risk factors, women, Pakistan	Pakistan

**Table 2:** Distribution of the abstracted risk factors across the corpus of reviews identified for qualitative synthesis.

**Article short name**	**Risk factors by degree of association per article**																								
	**[1]**	**[2]**	**[3]**	**[4]**	**[5]**	**[6]**	**[7]**	**[8]**	**[9]**	**[10]**	**[11]**	**[12]**	**[13]**	**[14]**	**[15]**	**[16]**	**[17]**	**[18]**	**[19]**	**[20]**	**[21]**	**[22]**	**[23]**	**[24]**	**[25]**
Banti et al. (34)	***	***	***		*					**	***	**	*		*	*		*	**						
Beck (35)	**		*	**		*			**	*	*	*					**			**	*	*			
Cutrona (32)	**	*				**	**		***	*	***	**			**						**		*		
Guintivano et al. (33)	***	***			***	*				*	***	***	**		***								***	**	**
Gulamani et al. (36)	*	*	*		*	*					*		**			**	**						*		
Koirala and Chuemchit (37)								**	***			***				**									
Lee and Chung (38)	**		*	*	*		*				*	*				*			*						
O'Hara and Swain (39)				***	**	***	**				***	*	*	*	*				**						
Özcan et al. (40)				**	***	***			**		***	***	*	***	***	*			*			***			*
Robertson et al. (41)	***		***	***							***	**	*		*				**						
Schmied et al. (42)			*	*		*	*		*		*	*	*		*							*			*
Takegata et al. (43)						**	**		**			**			*	***				*		*			
Yim et al. (44)						**	*	**	***		**	*			*	*	**				**				
Zahidie and Jamali (45)						**	**		**	**	**	**	**			***	**			*					**

Abstracted risk factors indicate degree of association to postnatal depression with asterisks (Weak = *; Moderate = **; Strong = ***).

*[1] Presence of depressive symptoms during pregnancy; [2] Presence of symptoms of common mental disorders other than depression and anxiety during pregnancy; [3] Prior diagnosis of a depressive disorder; [4] Prior diagnosis of an anxiety disorder including prenatal anxiety; [5] Family history of psychiatric illness during or prior to pregnancy including genetic risk factors; [6] Perceived low social support during pregnancy; [7] Perceived low support from partner; [8] History of childhood sexual abuse; [9] Exposure to traumatic events during or prior to pregnancy specifically including physical domestic and family violence; [10] Generalized high allostatic load including the stress hormone cortisol and plasma-derived inflammatory biomarkers; [11] Significant life events occurring during pregnancy or immediately post-partum (e.g., death of a loved one; loss of employment; relationship breakdown or divorce; relocation including moving house); [12] Marital dissatisfaction leading to complications (including psycho-emotional but not physical domestic and family violence); [13] Adverse obstetric factors (e.g., pre-eclampsia; hyperemesis; premature labor including Cesarean section; intrapartum bleeding; pre-term birth); [14] Severe neonatal complications including congenital malformations; [15] Low socioeconomic status (i.e., low average income and/or high cost-of-living); [16] Specific culture-bound factors (e.g., spousal disappointment with sex of fetus/infant; imposition of strict gender roles during and after pregnancy); [17] High stress associated foremost with care of index child but including other young children; [18] Failure to adhere to psychiatric medications including those prescribed to manage depressive symptoms; [19] High maternal neuroticism; [20] Low maternal self-esteem and/or self-acceptance; [21] Difficult infant temperament; [22] Ambivalence associated with parenting including unplanned pregnancy; [23] Historical diagnosis of other common mental disorders; [24] Adverse experiences associated with immigration (e.g., racial/ethnic discrimination, delayed visa status/uncertainty surrounding immigration status, poor access to health services, low language ability for country of settlement); [25] Giving birth at age extremes (i.e., very young or older mothers)*.

**Table 3:** Hypotheses for how the COVID-19 crisis may exacerbate known risk factors for postnatal depression.

**Established risk factor for postnatal depression (numbers listed in brackets are per Table 2)**	**Hypotheses: Exacerbated by COVID-19 crisis? *[strongly decreased, weakly decreased, N/A, weakly increased, strongly increased]***	**Examples of contextual factors that may be interacting with this risk factor**	**Disaster-related rationale for hypotheses**
[1] Presence of depressive symptoms during pregnancy	Strongly increased	Home isolation; social and physical distancing; some antenatal and postnatal supports have ceased operating; reduced physical activity; cumulative losses; increased media exposure.	Increased rates of depression symptoms experienced in populations following disasters (5), and currently observed in relation to COVID-19 (28, 29). Reduced rates of physical activity reported for those in the perinatal period during COVID-19 pandemic (29).
[2] Presence of symptoms of common mental disorders (other than depression and anxiety) during pregnancy	Weakly increased	Home isolation; social and physical distancing; some antenatal and postnatal supports have ceased operating; reduced physical activity; cumulative losses; increased media exposure.	Increased rates of psychopathology currently observed in relation to COVID-19 (28).
[3] Prior diagnosis of a depressive disorder	N/A for the cohort pregnant and/or giving birth during the COVID-19 pandemic		
[4] Prior diagnosis of an anxiety disorder including prenatal anxiety	Strongly increased	Home isolation; social and physical distancing; some antenatal and postnatal supports have ceased operating; reduced physical activity; cumulative losses; increased media exposure.	Increased rates of anxiety symptoms experienced in populations following disasters (5), and currently observed in relation to COVID-19 (28, 29).
[5] Family history of psychiatric illness, during or prior to pregnancy, including genetic risk factors	N/A for the cohort pregnant and/or giving birth during the COVID-19 pandemic		
[6] Perceived low social support during pregnancy	Strongly increased	Home isolation; social and physical distancing; reduced visitations from social supports; some postnatal supports have ceased operating; reduced time in hospital; reduced number/length of medical appointments.	Social support can alleviate the stress caused by disaster, however it appears to depend on whether support structures are created or destroyed (5). It appears that COVID-19 is likely to reduce the likelihood that social supports can be effectively accessed, thus perceived social support is likely to be lower.
[7] Perceived low support from partner	Weakly increased	Partners may be physically present in the home whilst working from home; possibly increased interpersonal partner conflict from containment in the home for long periods.	Social support can alleviate the stress caused by disaster, however it appears to depend on whether support structures are created or destroyed (5). It appears that COVID-19 may have mixed impacts regarding partner relationships with some partners more able to support when working from home, whereas other families may experience increased interpersonal partner conflict from containment in the home for long periods (17).
[8] History of childhood sexual abuse	N/A for the cohort pregnant and/or giving birth during the COVID-19 pandemic		
[9] Exposure to traumatic events during or prior to pregnancy specifically including physical domestic and family violence	Strongly increased	Home isolation; social and physical distancing; reduced visitations from social supports; changes in hospital policies, for instance, separation of COVID-19 positive mothers from their newborn infants for 14 days in China (9); no birth partners in the labor ward (e.g., in New York in the United States of America).	Domestic and family violence expected to increase during disaster, particularly the COVID-19 crisis (17). Additionally, for some women the impact of changed hospital policies in times of disaster may be perceived to be traumatic.
[10] General stress (i.e., Generalized high allostatic load including the stress hormone cortisol and plasma-derived inflammatory biomarkers)	Strongly increased	Home isolation; social and physical distancing; reduced visitations from social supports; some postnatal supports have ceased operating; reduced time in hospital; reduced number/length of medical appointments; media exposure; financial stress associated with employment uncertainty (e.g., loss of employment hours).	Emerging research from the COVID-19 crisis indicates high levels of stress and associated psychopathology in the general population (6), and high levels of stress have also been recorded within perinatal populations (28).
[11] Significant life events occurring during pregnancy or immediately post-partum (e.g., death of a loved one; loss of employment; relationship breakdown or divorce; relocation including moving house)	Strongly increased	COVID-19 may in itself be perceived as a significant life event; women may experience death of a loved one due to illness from COVID-19; women may not be able to mourn the death of a loved one in culturally expected ways due to imposed restrictions; loss of employment may be experienced for self or other family members; relationship strain from containment in the home for long periods may result in relationship breakdown or divorce.	No direct evidence identified from previous disasters.
[12] Marital dissatisfaction leading to complications (including psycho-emotional but not physical domestic and family violence)	Weakly increased	Partners may be physically present in the home due to working from home; possibly increased interpersonal partner conflict from containment in the home for long periods; unequal caring and/or home-schooling duties may increase dissatisfaction.	Positive social support from partners can reduce the stress caused by disaster, however not all partners provide positive social support (5). Interpersonal partner conflict and marital dissatisfaction may be amplified within some families (17).
[13] Adverse obstetric factors (e.g., pre-eclampsia; hyperemesis; premature labor including Cesarean section; intrapartum bleeding; pre-term birth)	N/A		
[14] Severe neonatal complications including congenital malformations	N/A		
[15] Low socioeconomic status (i.e., low average income and/or high cost-of-living)	Weakly increased	Loss of employment for self or other family members may change the experience of socio-economic well-being.	Economic factors, such as family income and employment, have been linked to poor maternal mental health after earthquakes (5).
[16] Specific culture-bound factors (e.g., spousal disappointment with sex of fetus/infant; imposition of strict gender roles during and after pregnancy)	N/A		
[17] High stress associated foremost with care of index child but including other young children	Strongly increased	Home isolation with reduced visitations from social supports may increase the perceived stress associated with the index child, and high stress may result from reduced care options (i.e., keeping other children home from care; home schooling; etc.).	No direct evidence identified from previous disasters.
[18] Failure to adhere to psychiatric medications including those prescribed to manage depressive symptoms	N/A		
[19] High maternal neuroticism	N/A		
[20] Low maternal self-esteem and/or self-acceptance	N/A		
[21] Difficult infant temperament	N/A		
[22] Ambivalence associated with parenting, including unplanned pregnancy	N/A		
[23] Historical diagnosis of other common mental disorders	N/A for the cohort pregnant and/or giving birth during the COVID-19 pandemic		
[24] Adverse experiences associated with immigration (e.g., racial/ethnic discrimination, delayed visa status/uncertainty surrounding immigration status, poor access to health services, low language ability for country of settlement)	Weakly increased	Physical distancing; change in economic climate.	Possible increased uncertainty surrounding immigration status with possible longer wait times; access to health services may be impacted; and ability to source help services without face-to-face interaction may be increasingly challenging.
[25] Giving birth at age extremes (i.e., very young or older mothers)	N/A		

## Discussion

From the 14 articles that were identified in this review (as shown in Table 1), it is evident that there are a range of risk factors that have been consistently found to increase the likelihood of women experiencing postnatal depression (as shown in Table 2). Although some of these risk factors are unlikely to be increased, others are more likely to be increased during and following the COVID-19 pandemic (see Table 3). In particular, it was identified that the following factors are at a strong risk of being exacerbated in the COVID-19 crisis: Presence of depressive symptoms during pregnancy; prior diagnosis of an anxiety disorder including prenatal anxiety; perceived low social support during pregnancy; exposure to traumatic events during or prior to pregnancy (specifically including physical, domestic, and family violence); stress levels (i.e., high generalized allostatic load); significant life events occurring during pregnancy or immediately post-partum (e.g., death of a loved one; loss of employment; relationship breakdown or divorce; relocation including moving house); high stress associated foremost with care of index child but including care of other children; and adverse experiences associated with immigration (e.g., racial/ethnic discrimination; delayed visa status/uncertainty surrounding immigration status; poor access to health services; low language ability for country of settlement). There is also a chance that the following factors may also impact women at this time and therefore need to be monitored in relation to postnatal depression rates: Presence of symptoms of common mental disorders (other than depression and anxiety) during pregnancy; perceived low support from partner; marital dissatisfaction leading to complications (including psycho-emotional but not physical domestic and family violence); low socioeconomic status (i.e., low average income and/or high cost-of-living) particularly in view of the economic shocks leading to reduced employment as a result of the COVID-19 crisis. Given the description of the current COVID-19 climate for pregnant women and the known risk factors that have been identified from previous review papers, it is further hypothesized that overall current population prevalence rates of postnatal depression will increase.

### Implications for Research, Practice, and Policy

It is therefore essential that researchers actively examine the identified factors that may increase postnatal depression risk in the context of a pandemic generally, and the COVID-19 pandemic specifically. We urge research funding bodies to work with researchers and mothers with lived experience of postnatal depression to ensure that this research priority is met. Advancing the limited knowledge base regarding maternal postnatal depression risk following crises, particularly pandemics, could improve future government and clinical decision-making. New cross-sectional and longitudinal cohort studies that attempt to recruit mothers in the perinatal period during the COVID-19 pandemic are therefore urgently needed. Additionally, it may be useful for researchers to consider designs, such as those implemented by Jiang et al. (28), where a previous cohort was used as a comparison group for understanding contextual changes and the impact on maternal and child well-being. Further, research is needed into which mitigation efforts are having a direct effect on population prevalence levels.

Given that postnatal depression is not a new phenomenon, there are effective ways to assess it clinically (e.g., Edinburgh Postnatal Depression Scale; Center for Epidemiologic Studies Depression Scale; Beck Depression Inventory), research and monitor community levels of postnatal depression, examine mechanisms that impact individuals' likely experience of postnatal depression, and intervene (47). For these reasons, healthcare workers (e.g., psychiatrists, midwives, general practitioners, psychologists, social workers, nurses) need to be aware of how known risk factors may be interacting in the context of the COVID-19 pandemic, and consider innovative ways that they can address mental health concerns during and following the COVID-19 pandemic. This is of particular concern as there may be larger numbers of women who could be experiencing postnatal depression due to the exacerbation of risk factors. There is support for the efficacy of telehealth to support caregiver well-being and parenting behavior, as well as internet-delivered psychological interventions for women in the antenatal and postnatal periods in reducing depressive symptoms [for a review and meta-analysis see Loughnan, Joubert (47)]. Yet it is possible that these interventions are not equally efficacious or accessible for all populations, particularly when technology access is not universal and there may be limitations to privacy (e.g., confinement-related crowding in the home). Health care workers also need to be aware of how structural barriers to noticing symptomology may interfere with identification of mothers experiencing postnatal depression at this time. For instance, telehealth options that do not include videos may place greater onus on mothers' self-reporting rather than allowing for additional visual cues to assist clinicians in identifying mothers who may be under-reporting symptoms.

Further, governments and funding bodies need to be aware of the increased need to fund research for monitoring community levels of postnatal depression and the costs involved in upscaling evidence-based interventions to meet increased demand. Research has shown that by investing in women's mental health during the perinatal period there is a reduction in long-term socio-emotional impacts, physical health, and associated societal/economic costs (48). Targeted funding at this critical time may contribute to reductions in the short- and long-term economic, physical health, and socio-emotional impacts for women and their children, feeding into economic recovery on a wider scale. Although there may be some amelioration of risks due to policy and clinical responses, we believe that as a net result there will be an increased number of women needing support for postnatal depression during and following the COVID-19 pandemic. Thus, critical to the success of upscaling evidence-based interventions is having a workforce that is capable and ready to implement them. It is therefore important to consider the training needs and/or digital adaptations that may need priority funding to ensure timely delivery and accessibility of evidence-based interventions to treat postnatal depression during and following the COVID-19 pandemic.

### Limitations and Strengths

This review is not without limitations. First, we were unable to use any risk of bias tools to examine study bias. This was due to a combination of factors including the heterogeneity of methods in the identified review papers, changes in reporting standards across the decades, and underreporting of core information in the identified review papers making it difficult to explicitly evaluate risk of bias. Second, it is possible that some risk factors for postnatal depression have not been captured by our review process. These may include risk factors that are important for specific populations, risk factors that may arise specifically in the context of the COVID-19 pandemic, as well as risk factors with a developing evidence-base that have not yet been captured in meta-analyses and peer-reviewed review papers. In addition, qualitative methodologies were employed to critically and clinically evaluate the likelihood that the risk factors identified by our process would be exacerbated by the COVID-19 crisis; as such, it is possible that alternative interpretations may be made. Further, we have attempted to identify those risk factors that may be impacted by a current contextual change, and have therefore excluded a range of early life experiences, genetic factors, and biological vulnerabilities to experience (44). While it may be possible that these risk factors will impact some women's risk of developing postnatal depression in a few decades, they are not likely to immediately impact current population prevalence rates of postnatal depression. Finally, an examination of resilience factors in relation to postnatal depression was beyond the scope of this review yet might be useful to consider alongside postnatal depression risk factors. Future research should consider eliciting resilience factors from previous research, as well as examining resilience factors that are specific to the COVID-19 pandemic.

Nonetheless, this review also has several noteworthy and timely strengths. Although, several studies have shown that stress during pregnancy and postpartum is associated with mental ill-health (4, 5), few researchers have been in the position to prospectively identify the mechanisms that may drive the association between experience of perinatal stress and mental ill-health. In this paper, we have outlined a number of ways that women in the perinatal period may be experiencing increased stress in the context of COVID-19. We have discussed findings from previous crises that indicate that women might be at a higher risk of developing postnatal depression in the wake of crises. Further, this review has highlighted several core risk factors for postnatal depression that are likely to be impacted and/or exacerbated by crisis contexts such as the COVID-19 global pandemic crisis. Thus, we believe that it is likely that there is a heightened chance that women are at risk of developing postnatal depression at this time, and that population rates of postnatal depression may be increased.

## Conclusion

It is essential for the research community to identify potential mechanisms underlying mental ill-health in crisis contexts so that assessment and testing can be prioritized, and policy makers can urgently address these mechanisms with emergency funding to ameliorate the effects of the COVID-19 pandemic on maternal mental health. In particular, researchers and policy makers should attempt to focus efforts on improving perceived social support, reducing exposure to traumatic events including physical domestic and family violence, reducing the impact of significant life events, and addressing the stress associated with caring for young children during a pandemic; as we hypothesize that these mechanisms may be particularly likely to drive change for women who are at risk of postnatal depression in the wake of the COVID-19 global pandemic crisis.

## Data Availability Statement

The original contributions presented in the study are included in the article, further inquiries can be directed to the corresponding author.

## Author Contributions

FD provided the study concept and design. The literature search was conducted by LK in close consultation with FD. FD interpreted the data. FD and LK drafted the manuscript and engaged in critical revision for important intellectual content. All authors contributed to the article and approved the submitted version.

## Conflict of Interest

The authors declare that the research was conducted in the absence of any commercial or financial relationships that could be construed as a potential conflict of interest.

## References

[1] Yao H Chen J-H Xu Y-F. Patients with mental health disorders in the COVID-19 epidemic. Lancet Psychiatr. (2020) 7:e21. 10.1016/S2215-0366(20)30090-0 PMC7269717 32199510

[2] Meaney MJ. Perinatal maternal depressive symptoms as an issue for population health. Am J Psychiatr. (2018) 175:1084–93. 10.1176/appi.ajp.2018.17091031 30068258

[3] World Health Organization. Mental Health Aspects of Women's Reproductive Health: A Global Review of the Literature. Geneva, Switzerland: WHO Press, World Health Organization (2009).

[4] Harville E Xiong X Buekens P. Disasters and perinatal health: a systematic review. Obstetrical Gynecol Survey. (2010) 65:713. 10.1097/OGX.0b013e31820eddbe 21375788 PMC3472448

[5] Ren J-H Chiang C-LV Jiang X-L Luo B-R Liu X-H Pang M-C. Mental disorders of pregnant and postpartum women after earthquakes: a systematic review. Disaster Med Public Health Prepared. (2014) 8:315–25. 10.1017/dmp.2014.62 25098648

[6] Wang C Pan R Wan X Tan Y Xu L Ho CS . Immediate psychological responses and associated factors during the initial stage of the 2019 coronavirus disease (COVID-19) epidemic among the general population in China. Int J Environ Res Public Health. (2020) 17:1729. 10.3390/ijerph17051729 32155789 PMC7084952

[7] Dias CC Figueiredo B. Breastfeeding and depression: a systematic review of the literature. J Affective Disord. (2015) 171:142–54. 10.1016/j.jad.2014.09.022 25305429

[8] Hermann A Deligiannidis KM Bergink V Monk C Fitelson EM Robakis TK . Response to SARS-Covid-19-Related Visitor Restrictions on Labor and Delivery Wards in New York City. Archives of Women's Mental Health (2020). p. 1–2. 10.1007/s00737-020-01030-2 PMC7156902 32296947

[9] Wang L Shi Y Xiao T Fu J Feng X Mu D . Chinese expert consensus on the perinatal and neonatal management for the prevention and control of the 2019 novel coronavirus infection. Annal Transl Med. (2020) 8:47. 10.21037/atm.2020.02.20 32154287 PMC7036629

[10] Agnafors S Bladh M Svedin CG Sydsjö G. Mental health in young mothers, single mothers and their children. BMC Psychiatr. (2019) 19:112. 10.1186/s12888-019-2082-y 30975129 PMC6460673

[11] Collins CH Zimmerman C Howard LM. Refugee, asylum seeker, immigrant women and postnatal depression: rates and risk factors. Arch Women's Mental Health. (2011) 14:3–11. 10.1007/s00737-010-0198-7 21153849

[12] Falah-Hassani K Shiri R Vigod S Dennis C-L. Prevalence of postpartum depression among immigrant women: a systematic review and meta-analysis. J Psychiatr Res. (2015) 70:67–82. 10.1016/j.jpsychires.2015.08.010 26424425

[13] Fellmeth G Fazel M Plugge E. Migration and perinatal mental health in women from low-and middle-income countries: a systematic review and meta-analysis. BJOG. (2017) 124:742–52. 10.1111/1471-0528.14184 27320110

[14] Rees SJ Fisher JR Steel Z Mohsin M Nadar N Moussa B . Prevalence and risk factors of major depressive disorder among women at public antenatal clinics from refugee, conflict-affected, and Australian-born backgrounds. JAMA Network Open. (2019) 2:e193442. 10.1001/jamanetworkopen.2019.3442 31050785 PMC6503483

[15] Shelton BA. Gender and Unpaid Work. Handbook of the Sociology of Gender. Boston, MA: Springer US (2006). p. 375–90. 10.1007/0-387-36218-5_17

[16] Bacchus L Mezey G Bewley S. Domestic violence: prevalence in pregnant women and associations with physical and psychological health. Eur J Obstetr Gynecol Reproduct Biol. (2004) 113:6–11. 10.1016/S0301-2115(03)00326-9 15036702

[17] Campbell AM. An Increasing Risk of Family Violence During the Covid-19 Pandemic: Strengthening Community Collaborations to Save Lives. Forensic Science International: Reports: 100089. 10.1016/j.fsir.2020.100089 PMC7152912 38620174

[18] Saulnier DD Brolin K. A systematic review of the health effects of prenatal exposure to disaster. Int J Public Health. (2015) 60:781–7. 10.1007/s00038-015-0699-2 26298438

[19] Zahran S Shelley TOC Peek L Brody SD. Natural disasters and social order: modeling crime outcomes in Florida. Int J Mass Emerg Disasters. (2009) 27:26–52. Available online at: http://www.ijmed.org

[20] Parkinson D. Investigating the increase in domestic violence post disaster: an Australian case study. J Int Violence. (2019) 34:2333–62. 10.1177/0886260517696876 29294681

[21] Schumacher JA Coffey SF Norris FH Tracy M Clements K Galea S. Intimate partner violence and Hurricane Katrina: predictors and associated mental health outcomes. Viol Victims. (2010) 25:588–603. 10.1891/0886-6708.25.5.588 21061866 PMC3394178

[22] Weitzman A Behrman JA. Disaster, disruption to family life, and intimate partner violence: the case of the 2010 earthquake in Haiti. Sociol Sci. (2016) 3:167–89. 10.15195/v3.a9

[23] Anastario M Shehab N Lawry L. Increased gender-based violence among women internally displaced in Mississippi 2 years post-Hurricane Katrina. Disaster Med Public Health Preparedness. (2009) 3:18–26. 10.1097/DMP.0b013e3181979c32 19293740

[24] Schwartz DA. An analysis of 38 pregnant women with COVID-19, their newborn infants, and maternal-fetal transmission of SARS-CoV-2: maternal coronavirus infections and pregnancy outcomes. Arch Pathol Lab Med. (2020) 144:799–805. 10.5858/arpa.2020-0901-SA 32180426

[25] Yang H Wang C Poon L. Novel coronavirus infection and pregnancy. Ultrasound Obstetr Gynecol. (2020) 55:435. 10.1002/uog.22006 PMC7169856 32134165

[26] Rasmussen SA Jamieson DJ Uyeki TM. Effects of influenza on pregnant women and infants. Am J Obstetr Gynecol. (2012) 207:S3–8. 10.1016/j.ajog.2012.06.068 22920056

[27] Silasi M Cardenas I Kwon JY Racicot K Aldo P Mor G. Viral infections during pregnancy. Am J Reproduct Immunol. (2015) 73:199–213. 10.1111/aji.12355 25582523 PMC4610031

[28] Jiang H Jin L Qian X Xiong X La X Chen W . Evidence of accessing antenatal care information via social media platforms supports mental wellbeing in COVID-19 epidemic. Bull World Health Organ. (2020). 10.2471/BLT.20.255489

[29] Davenport MH Meyer S Meah VL Strynadka MC Khurana R. Moms are not OK: COVID-19 and Maternal Mental Health. Front Glob Women's Health. (2020) 86:140–6. 10.3389/fgwh.2020.00001 PMC8593957 34816146

[30] Moher D Liberati A Tetzlaff J Altman DG Group P. Preferred reporting items for systematic reviews and meta-analyses: the PRISMA statement. PLoS Med. (2009) 6:e1000097. 10.1371/journal.pmed.1000097 19621072 PMC2707599

[31] Downs SH Black N. The feasibility of creating a checklist for the assessment of the methodological quality both of randomised and non-randomised studies of health care interventions. J Epidemiol Commun Health. (1998) 52:377–84. 10.1136/jech.52.6.377 9764259 PMC1756728

[32] Cutrona CE. Nonpsychotic postpartum depression: a review of recent research. Clin Psychol Rev. (1982) 2:487–503. 10.1016/0272-7358(82)90026-5

[33] Guintivano J Sullivan P Stuebe A Penders T Thorp J Rubinow D . Adverse life events, psychiatric history, and biological predictors of postpartum depression in an ethnically diverse sample of postpartum women. Psychol Med. (2018) 48:1190–200. 10.1017/S0033291717002641 28950923 PMC6792292

[34] Banti S Borri C Camilleri V Cortopassi C Montagnani MS Ramacciotti D . Perinatal mood and anxiety disorders. Clinical assessment and management. A review of current literature. Giorn Ital Psicopat. (2009):351–66. 10.1016/j.nurpra.2018.03.010

[35] Beck CT. Postpartum depression: a metasynthesis. Qual Health Res. (2002) 12:453–72. 10.1177/104973202129120016 11939248

[36] Gulamani SS Premji SS Kanji Z Azam SI. A review of postpartum depression, preterm birth, and culture. J Perinatal Neonatal Nursing. (2013) 27:52–9. 10.1097/JPN.0b013e31827fcf24 23360942

[37] Koirala P Chuemchit M. Depression and domestic violence experiences among Asian women: a systematic review. Int J Women's Health. (2020) 12:21. 10.2147/IJWH.S235864 32021490 PMC6970613

[38] Lee DT Chung TK. Postnatal depression: an update. Best Pract Res Clin Obstetr Gynaecol. (2007) 21:183–91. 10.1016/j.bpobgyn.2006.10.003 17157072

[39] O'Hara MW Swain AM. Rates and risk of postpartum depression-a meta-analysis. Int Rev Psychiatr. (1996) 8:37–54. 10.3109/09540269609037816

[40] Özcan NK Boyacioglu NE Dinç H. Postpartum depression prevalence and risk factors in Turkey: a systematic review and meta-analysis. Arch Psychiatr Nursing. (2017) 31:420–8. 10.1016/j.apnu.2017.04.006 28693880

[41] Robertson E Grace S Wallington T Stewart DE. Antenatal risk factors for postpartum depression: a synthesis of recent literature. General Hospital Psychiatr. (2004) 26:289–95. 10.1016/j.genhosppsych.2004.02.006 15234824

[42] Schmied V Johnson M Naidoo N Austin M-P Matthey S Kemp L . Maternal mental health in Australia and New Zealand: a review of longitudinal studies. Women Birth. (2013) 26:167–78. 10.1016/j.wombi.2013.02.006 23583667

[43] Takegata M Ohashi Y Lazarus A Kitamura T editors. Cross-national differences in psychosocial factors of perinatal depression: a systematic review of India and Japan. Healthcare. (2017) Multidisciplinary Digital Publishing Institute. 10.3390/healthcare5040091 PMC5746725 29207561

[44] Yim IS Stapleton LRT Guardino CM Hahn-Holbrook J Schetter CD. Biological and psychosocial predictors of postpartum depression: systematic review and call for integration. Ann Rev Clin Psychol. (2015) 11:99–137. 10.1146/annurev-clinpsy-101414-020426 25822344 PMC5659274

[45] Zahidie A Jamali T. An overview of the predictors of depression among adult Pakistani women. J College Phys Surg Pakistan. (2013) 23:574–80. 23930875

[46] Cohen J. Statistical Power Analysis for the Behavioral Sciences. 2nd ed. Hillsdale, NJ: Erbaum Press (1988).

[47] Loughnan SA Joubert AE Grierson A Andrews G Newby JM. Internet-delivered psychological interventions for clinical anxiety and depression in perinatal women: a systematic review and meta-analysis. Arch Women's Mental Health. (2019) 2:737–50. 10.1007/s00737-019-00961-9 31101993

[48] Pham CT Karnon JD Middleton PF Bloomfield FH Groom KM Crowther CA . Randomised clinical trials in perinatal health care: a cost-effective investment. Med J Australia. (2017) 207:289–93. 10.5694/mja16.01178 28954615

